# Anti-Swing Control for Quadrotor-Slung Load Transportation System with Underactuated State Constraints

**DOI:** 10.3390/s23218995

**Published:** 2023-11-06

**Authors:** Feng Ding, Chong Sun, Shunfan He

**Affiliations:** Hubei Provincial Engineering Research Center for Intelligent Management of Manufacturing Enterprises, School of Computer Science, South-Central Minzu University, Wuhan 430074, China; dingfeng@mail.scuec.edu.cn (F.D.); heshunfan@mail.scuec.edu.cn (S.H.)

**Keywords:** quadrotor-slung load transportation system, barrier Lyapunov method, state constraints, underactuated system

## Abstract

Quadrotors play a crucial role in the national economy. The control technology for quadrotor-slung load transportation systems has become a research hotspot. However, the underactuated load’s swing poses significant challenges to the stability of the system. In this paper, we propose a Lyapunov-based control strategy, to ensure the stability of the quadrotor-slung load transportation system while satisfying the constraints of the load’s swing angles. Firstly, a position controller without swing angle constraints is proposed, to ensure the stability of the system. Then, a barrier Lyapunov function based on the load’s swing angle constraints is constructed, and an anti-swing controller is designed to guarantee the states’ asymptotic stability. Finally, a PD controller is designed, to drive the actual angles to the virtual ones, which are extracted from the position controller. The effectiveness of the control method is verified by comparing it to the results of the LQR algorithm. The proposed control method not only guarantees the payload’s swing angle constraints but also reduces energy consumption.

## 1. Introduction

With their versatility, agility, and maneuverability, quadrotors have applications spanning multiple industries, such as search and rescue, aerial photography and videography, and agriculture. They play an important role in our daily lives. However, the challenge of ensuring stability in the quadrotor remains a critical issue because it is inherently underactuated, with multivariables. In recent decades, much work has been done on the control of quadrotors [1,2,3,4,5]. An inner–outer control structure is the most common strategy, in which the inner loop is the attitude subsystem and the outer one is the position subsystem.

With the potential application of quadrotors, the control technology for quadrotor transportation systems has become a popular research topic. Some researchers focus on the transportation system, where the payload is fixed rigidly at the bottom of the quadrotor [6,7]. However, when the load’s center of gravity deviates from the system’s central axis, it alters the inertia of the transportation system. This can render the attitude controller ineffective, due to the inaccurate dynamics. In recent years, a quadrotor transportation system called the quadrotor-slung load transportation system (QSLTS), where a load hangs on the quadrotor by a flexible cable, has attracted much attention [8,9]. In such a system, the swing of the payload does not affect the inertia of the system, but the position dynamics are underactuated. Accordingly, the swing of the load presents many challenges to the stability of the quadrotor. Much attention has been given to this issue.

The control of the QSLTS initially focused on the study of the planar quadrotor, neglecting the rotation of the quadrotor and assuming that the payload swing was on the same plane. Therefore, the research results only had theoretical guidance significance [10]. In the control of the QSLTS, the main task of the quadrotor is to transport the payload to the expected location safely. Therefore, some control methods regard the payload as a disturbance and design robust controllers to position the quadrotor [11,12]. However, the payload’s swing not only poses a significant challenge to the stability of the quadrotor but also can lead to the risk of collision and damage to fragile payloads. To address this issue, the design of the controller should aim to reduce the payload’s swing. An effective strategy is to regard the payload and quadrotor as a whole, establishing a quadrotor–payload dynamic model, and designing a controller to achieve quadrotor positioning while swiftly suppressing the payload’s vibration [13,14]. This approach considers the issue of swing angle suppression during controller design. Yang [15] proposed an energy-based nonlinear controller, to ensure the position of the quadrotor and the swing angle payload asymptotically. Trajectory planning is one of the commonly used methods to solve state constraint problems [16,17,18]. Alkomy [19] conducted a comparison of several polynomial trajectories, to determine which one leads to less vibration. Liang X proposed optimal time-based motion trajectory planning for the QSLTS under constraint states [20]. However, the robustness of the system could not be guaranteed.

Some work has been done to attenuate the swing angles of the payload, but the effect has not been quantitatively evaluated. Therefore, the constraints of the payload’s swing should be taken into account during the design of the position controller. The rapid positioning of the quadrotor while suppressing the payload’s swing is essentially the control of an underactuated system with multiple degrees of freedom (DoF) under state constraints. However, this issue is still an ongoing research topic [21]. Various control strategies, such as the barrier Lyapunov method [22,23,24,25], model predictive control [26,27], adaptive fuzzy control [28,29], and the neuroadaptive learning algorithm [30] have been proposed for state constraint systems. Among these methods, the barrier Lyapunov method exhibits excellent performance on state constraint limitation while ensuring the robustness of the controller. It has been applied in various systems, such as high-order nonlinear systems [22,23], variant unmanned aerial vehicles [24], and quadrotor UAVs [25]. However, the existing methods mainly focus on fully actuated systems with output constraints [31] and full-state constraints [32]. Few of these methods can be directly applied to underactuated systems. It is a challenge to design controllers for underactuated states because they lack independent inputs.

Based on the above discussion, there are some control challenges for the QSLTS.

1. Underactuated state constraints: To maintain the stability of the quadrotor and the safety of the payload itself, it is necessary to manage the swing of the payload. However, the swing angles lack independent control inputs, making it challenging to design controllers directly to address this issue. Although some open-loop controllers are designed according to trajectory planning, the robustness of the system cannot be guaranteed [20].
2. Control accuracy: Some control strategies [11,24,33,34] for nonlinear systems can only achieve uniformly ultimately bounded results, rather than asymptotic stability. As a result, the control accuracy cannot be assured.

Motivated by the above discussions, an anti-swing controller for the QSLTS, based on the barrier Lyapunov function, is proposed in this paper. The dynamics of the system are derived by regarding quadrotor and cable-suspended payload as a unified entity. An inner–outer control strategy is utilized, where the outer controller functions as an anti-swing controller, ensuring the stability of the underactuated position subsystem while limiting the swing angles in boundaries, while the inner one serves as an attitude controller, tracking the attitude angles extracted from the outer controller to guarantee the effectiveness the inner controller. Specifically, the dynamics of the position subsystem are reconstructed in a cascade form. Then, a position controller based on the Lyapunov function is proposed, to ensure asymptotic convergence for the subsystem without constraints. Furthermore, an anti-swing controller is designed, based on the barrier Lyapunov function, to guarantee the states’ asymptotic stability while the swing angles are limited in the boundaries. Finally, we construct a controller for the attitude subsystem, to drive the actual angles to the virtual ones.

The main contributions of our work include:

1. A Lyapunov-based controller is designed to guarantee the stability of the QSLTS while limiting the swing of the payload.
2. The states of the QSTLS have asymptotic stability, instead of being only uniformly ultimately bounded.

The rest of this paper is organized as follows: The preliminaries and problem formulation are presented in Section 2. Also in this section, the dynamics of the underactuated system are described. Then, controllers and their corresponding stability analysis are given in Section 3. Comparative simulation results and analysis are given in Section 4, to verify the effectiveness of the proposed methods, followed by a short conclusion in Section 5.

## 2. Preliminaries and Problem Statement

**Figure 1:**
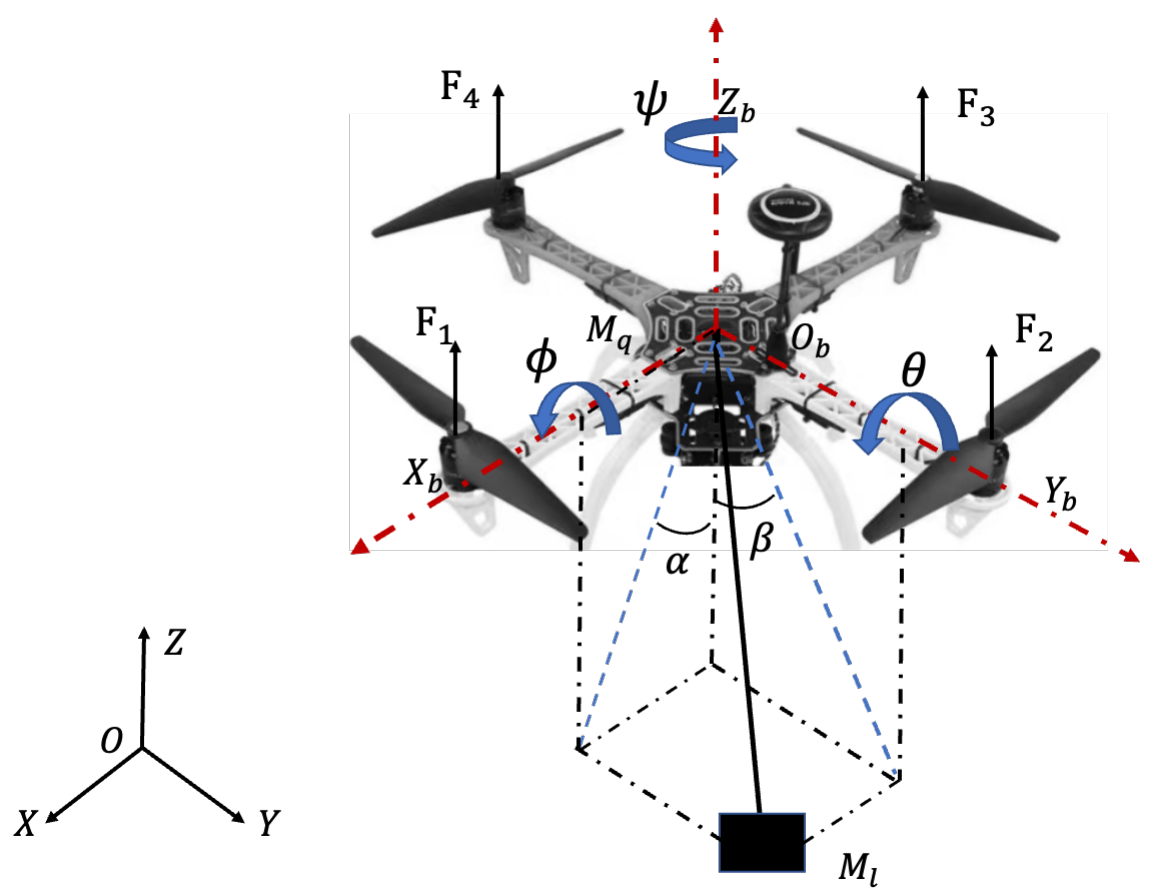
The structure and coordinates of the quadrotor-slung load transportation system.

The control problem of a transportation system where a payload hangs on a quadrotor directly is discussed in this paper. The schematic diagram and coordinate frames of the QSLTS are illustrated in Figure 1 and the symbols in the figure are shown in Table 1. The quadrotor is considered as a rigid and symmetrical body. The payload is suspended below the quadrotor directly by a flexible cable. Let $\xi ={\left[x,y,z\right]}^{T}\in R^{3\times 1}$ be the position vector in the generalized coordinate. The rotation vector of the quadrotor in the Euler coordinate system is $\Phi ={\left[\phi ,\theta ,\psi \right]}^{T}\in R^{3\times 1}$, where ϕ,θ,ψ are the roll angle, pitch angle, and yaw angle, respectively.

We also assume that the cable is tense while the quadrotor is moving and that the swing angles satisfy |α|<π/2,β<π/2.

The position subsystem of the QSLTS can be constructed as an underactuated model based on the Lagrange equation [35]:

(1) $$\begin{array}{c} \left[\begin{array}{ccc} m_{11} & m_{12} & m_{13}\\ m_{21} & m_{22} & m_{23}\\ m_{31} & m_{32} & m_{33} \end{array}\right]\left[\begin{array}{c} {\ddot{\xi }}_{1}\\ {\ddot{\xi }}_{2}\\ \ddot{\xi_{3}} \end{array}\right]+\left[\begin{array}{c} C_{1}\\ C_{2}\\ C_{3} \end{array}\right]+\left[\begin{array}{c} 0_{2\times 1}\\ 0\\ G \end{array}\right]=\left[\begin{array}{c} U_{xy}\\ U_{z}\\ 0_{2\times 1} \end{array}\right] \end{array}.$$

The attitude dynamics can be constructed:

(2) $$\begin{array}{c} I_{x}\ddot{\phi }=\left(I_{y}-I_{z}\right)\dot{\theta }\dot{\psi }+\tau_{x}\\ I_{y}\ddot{\theta }=\left(I_{z}-I_{x}\right)\dot{\phi }\dot{\psi }+\tau_{y}\\ I_{z}\ddot{\psi }=\left(I_{x}-I_{y}\right)\dot{\phi }\dot{\theta }+\tau_{z}, \end{array}$$

where $\xi_{1}$=${\left[x\;y\right]}^{T}$, $\xi_{2}=z$, $\xi_{3}$=${\left[\alpha \;\beta \right]}^{T}$, $U_{xy}={\left[U_{x},U_{y}\right]}^{T}$ and $U_{z}$ are the forces acting on the quadrotor along the X, Y, and Z axes, respectively.

$$\begin{array}{c} \;m_{11}=\left[\begin{array}{cc} M_{q}+M_{l} & 0\\ 0 & M_{q}+M_{l} \end{array}\right],m_{12}=\left[\begin{array}{c} 0\\ 0 \end{array}\right],m_{13}=M_{l}l\left[\begin{array}{cc} C_{\alpha }C_{\beta } & -S_{\alpha }S_{\beta }\\ 0 & C_{\beta } \end{array}\right],\\ \;m_{31}=\left[\begin{array}{cc} C_{\alpha } & 0\\ -S_{\alpha }S_{\beta } & C_{\beta } \end{array}\right],m_{32}=\left[\begin{array}{c} S_{\alpha }\\ C_{\alpha }S_{\beta } \end{array}\right],m_{33}=l\left[\begin{array}{cc} C_{\beta } & 0\\ 0 & 1 \end{array}\right],m_{23}=M_{l}l{\left[\begin{array}{c} S_{\alpha }C_{\beta }\\ C_{\alpha }S_{\beta } \end{array}\right]}^{T}, \end{array}$$

$$\begin{array}{c} \;C_{1}=M_{l}l\left[\begin{array}{c} -S_{\alpha }C_{\beta }{\dot{\alpha }}^{2}-S_{\alpha }C_{\beta }{\dot{\beta }}^{2}-2C_{\alpha }S_{\beta }\dot{\alpha }\dot{\beta }\\ -S_{\beta }{\dot{\beta }}^{2} \end{array}\right],C_{3}=l\left[\begin{array}{c} -2S_{\beta }\dot{\alpha }\dot{\beta }\\ C_{\beta }S_{\beta }{\dot{\alpha }}^{2} \end{array}\right],G=g\left[\begin{array}{c} S_{\alpha }\\ C_{\alpha }S_{\beta } \end{array}\right],\\ \;m_{21}=m_{12}^{T},m_{22}=M_{q}+M_{l},C_{2}=M_{l}l\left(C_{\alpha }C_{\beta }{\dot{\alpha }}^{2}+C_{\alpha }C_{\beta }{\dot{\beta }}^{2}-2S_{\alpha }S_{\beta }\dot{\alpha }\dot{\beta }\right)\\ U_{xy}=\left[\begin{array}{c} F(S_{\theta }C_{\phi }C_{\psi }+S_{\phi }S_{\psi })\\ F(S_{\theta }C_{\phi }S_{\psi }-S_{\phi }C_{\psi }) \end{array}\right],U_{z}=FC_{\theta }C_{\phi }-\left(M_{q}+M_{l}\right)g. \end{array}$$

$S_{*},C_{*}$ represent sin(∗) and cos(∗), respectively. $F=\Sigma ∥F_{i}∥$ is the resultant of the force generated by the rotors; $\tau_{x}$, $\tau_{y}$, and $\tau_{z}$ are the control torque related to the rotor speed [36]:

$$\left[\begin{array}{c} F\\ \tau_{x}\\ \tau_{y}\\ \tau_{z} \end{array}\right]=\left[\begin{array}{cccc} k_{f} & k_{f} & k_{f} & k_{f}\\ k_{f}l_{f} & 0 & -k_{f}l_{f} & 0\\ 0 & k_{f}l_{f} & 0 & -k_{f}l_{f}\\ k_{\tau } & -k_{\tau } & k_{\tau } & -k_{\tau } \end{array}\right]\left[\begin{array}{c} \omega_{1}^{2}\\ \omega_{2}^{2}\\ \omega_{3}^{2}\\ \omega_{4}^{2} \end{array}\right],$$

where $k_{f},k_{\tau }$ are aerodynamic coefficients and $\omega_{i}\left(i=1\cdots 4\right)$ is the rotational speed of the *i*th rotor. The distance between the rotor and the mass center of the quadrotor is denoted by $l_{f}$.

Evidently, $m_{31}$ is invertible. New variables are defined as

$$q_{1}=\xi_{1}+\int m_{31}^{-1}m_{32}{\dot{\xi }}_{2}dt+\int m_{31}^{-1}m_{33}{\dot{\xi }}_{3}dt,q_{2}=m_{31}{\dot{\xi }}_{1}+m_{32}{\dot{\xi }}_{2}+m_{33}{\dot{\xi }}_{3},q_{3}=\xi_{3},q_{4}={\dot{\xi }}_{3},q_{5}=\xi_{2},q_{6}={\dot{\xi }}_{2}.$$

Based on feedback linearization theory, we transform (1) into a combination of a cascade form of an underactuated subsystem and an actuated subsystem:

(3) $$\begin{array}{c} {\dot{q}}_{1}=m_{31}^{-1}q_{2}\\ {\dot{q}}_{2}=f_{1}+f_{2}\\ {\dot{q}}_{3}=q_{4}\\ {\dot{q}}_{4}=g_{1}+b_{1}u\\ {\dot{q}}_{5}=q_{5}\\ {\dot{q}}_{6}=g_{2}+b_{2}u, \end{array}$$

where $q_{1}\in R^{2\times 1}$ is underactuated, $q_{3}\in R^{2\times 1},q_{5}\in R^{1}$ are actuated, and $f_{2}=-C_{3}+{\dot{m}}_{31}{\dot{\xi }}_{1}+{\dot{m}}_{32}{\dot{\xi }}_{2}+{\dot{m}}_{33}{\dot{\xi }}_{3}$ is the cross item which can be ignored in the controller design; $g_{1}\in R^{2\times 1},g_{2}\in R^{1},b_{1}\in R^{2\times 3}$ and $b_{2}\in R^{1\times 3}$ can be derived from the matrix calculation of (1).

## 3. Controller Design

**Figure 2:**
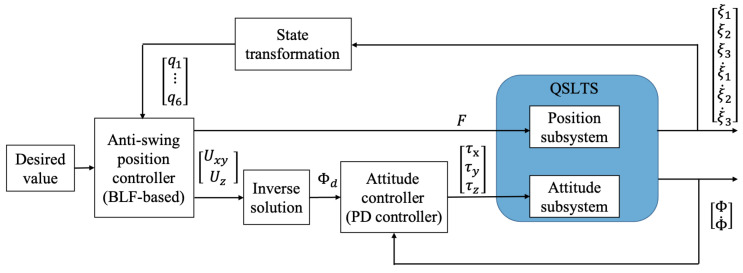
The control structure of the QSLTS.

There are two objectives for the quadrotor while transporting: flying to the expected position accurately and suppressing the payload’s swing effectively. We transform the anti-swing issue into a balance control problem. By determining the equilibrium of the payload directly beneath the quadrotor, an inner–outer control structure is proposed, to guarantee the stability of the QSLTS, where the outer loop is the position controller and the inner loop is the attitude controller. Specifically, an anti-swing controller is designed for the position subsystem to locate the quadrotor while eliminating the payload’s swing angles. Then, a PD controller is designed for the attitude subsystem to ensure the actual force to track the virtual output of the position controller. The control structure is illustrated in Figure 2.

### 3.1. Position Controller Based on Lyapunov Method

Based on the new variables, we define the following errors:

$$e_{1}=q_{1}-q_{1d},e_{2}=q_{2}-q_{2d},e_{3}=f_{1}-f_{1d},e_{4}={\dot{f}}_{1}-{\dot{f}}_{1d},e_{5}=q_{5}-q_{5d},q_{6}=x_{6}-x_{6d},$$

where $q_{1d},q_{2d},f_{d},{\dot{f}}_{d}$, $q_{5d}$, and $q_{6d}$ denote the desired value of $q_{1},q_{2},f,\dot{f}$, $q_{5}$, and $q_{6}$, respectively; $q_{2d}=f_{d}={\dot{f}}_{d}=0$, $q_{6d}=0$.

We design virtual controllers, $\lambda_{i}=-k_{i}z_{i}$, where $k_{i}\in R^{2\times 2}$ is a positive diagonal matrix, i=1,2,3, and $\lambda_{4}=-k_{4}z_{5}$, where $k_{4}\in R^{1}$ is a positive constant.

We choose new errors:

$$z_{1}=e_{1},z_{2}=e_{2}-\lambda_{1},z_{3}=e_{3}-\lambda_{2},z_{4}=e_{4}-\lambda_{3},z_{5}=e_{5},z_{6}=e_{6}-\lambda_{4}.$$

**Theorem** **1.** *Considering the QSLTS (1) without state constraints, a position controller based on the Lyapunov method is designed. When a set of control parameters (5) is satisfied, the controller (4) guarantees the states’ global asymptotic exponential stability:*

(4) $$u=-{\left[\begin{array}{c} \frac{\partial f_{1}}{\partial q_{3}}b_{1}\\ b_{2} \end{array}\right]}^{-1}\left[\begin{array}{c} u_{1}\\ u_{2} \end{array}\right],$$

where

$$\begin{array}{c} u_{1}=\left(\frac{d}{dt}\frac{\partial f_{1}}{\partial q_{3}}\right)q_{4}+\frac{\partial f_{1}}{\partial q_{3}}g_{1}+\vartheta_{1}z_{4}+\left(I+k_{3}k_{2}-k_{3}k_{3}\right)z_{3}+k_{2}k_{3}\left(k_{1}m_{31}^{-1}-k_{2}\right)z_{2}\\ -k_{3}k_{2}k_{1}m_{31}^{-1}k_{1}z_{1}\\ u_{2}=g_{2}+\vartheta_{2}z_{6}+\left(I-k_{4}k_{4}\right)z_{5}, \end{array}$$

where $\frac{\partial f_{1}}{\partial q_{3}}=g\left[\begin{array}{cc} C_{\alpha } & 0\\ -S_{\alpha }S_{\beta } & C_{\alpha }C_{\beta } \end{array}\right]$.

We choose proper parameters to satisfy

(5) $$\left\{\begin{array}{c} k_{i}>0,i=1,\cdots ,4\\ \vartheta_{1}>k_{3},\vartheta_{2}>k_{4}\\ eig(Q_{j})>0,j=1,\cdots ,6, \end{array}\right.$$

where

(6) $$\left\{\begin{array}{c} Q_{1}=m_{31}^{-1}k_{1}-0.25K_{1}^{T}K_{1}-0.25K_{3}^{T}K_{3}\\ Q_{2}=k_{2}-k_{1}m_{31}^{-1}-1.25\\ Q_{3}=k_{3}-k_{2}-K_{2}^{T}K_{2}-1\\ Q_{4}=\vartheta_{1}-k_{3}\\ Q_{5}=k_{4}\\ Q_{6}=\vartheta_{2}-k_{4}\\ K_{1}=m_{31}^{-1}-k_{1}m_{31}^{-1}k_{1}\\ K_{2}=I+k_{2}k_{1}m_{31}^{-1}-k_{2}k_{2}\\ K_{3}=k_{2}k_{1}m_{31}^{-1}k_{1}. \end{array}\right.$$

**Proof** **of** **Theorem** **1.** We choose the Lyapunov function

(7) $$V_{1}=\frac{1}{2}\sum_{j=1}^{6}z_{j}^{T}z_{j}.$$

Deriving both sides of (7), we obtain

$$\begin{array}{c} {\dot{V}}_{1}=z_{1}^{T}m_{31}^{-1}\left(z_{2}-k_{1}z_{1}\right)+z_{2}^{T}\left(z_{3}-k_{2}z_{2}-{\dot{\lambda }}_{1}\right)+z_{3}^{T}\left(z_{4}-k_{3}z_{3}-{\dot{\lambda }}_{2}\right)+z_{4}^{T}\left({\dot{e}}_{4}-{\dot{\lambda }}_{3}\right)\\ +z_{5}\left(z_{6}-k_{4}z_{5}\right)+z_{6}\left({\dot{e}}_{6}-{\dot{\lambda }}_{4}\right). \end{array}$$

According to the definition of $\lambda_{i}$, we obtain

(8) $$\left\{\begin{array}{c} {\dot{\lambda }}_{1}=-k_{1}m_{31}^{-1}\left(z_{2}-k_{1}z_{1}\right)\\ {\dot{\lambda }}_{2}=-k_{2}\left(z_{3}-k_{2}z_{2}+k_{1}m_{31}^{-1}\left(z_{2}-k_{1}z_{1}\right)\right)\\ {\dot{\lambda }}_{3}=-k_{3}\left(z_{4}-k_{3}z_{3}+k_{2}\left(z_{3}-k_{2}z_{2}+k_{1}m_{31}^{-1}\left(z_{2}-k_{1}z_{1}\right)\right)\right)\\ {\dot{\lambda }}_{4}=-k_{4}\left(z_{6}-k_{4}z_{5}\right). \end{array}\right.$$

Substituting (8) by (7), we obtain

$$\begin{array}{c} {\dot{V}}_{1}=-Z^{T}KZ+z_{1}^{T}\left(m_{31}^{-1}-k_{1}m_{31}^{-1}k_{1}\right)z_{2}+z_{2}^{T}\left(I+k_{2}k_{1}m_{31}^{-1}-k_{2}^{T}k_{2}\right)z_{3}\\ -z_{1}^{T}k_{2}k_{1}m_{31}^{-1}k_{1}z_{3}+z_{4}^{T}\left(\left(\frac{d}{dt}\frac{\partial f_{1}}{\partial q_{3}}\right)q_{4}+\frac{\partial f_{1}}{\partial q_{3}}\left(g_{1}+b_{1}u\right)+\left(I+k_{3}k_{2}-k_{3}k_{3}\right)z_{3}\right.\\ \left.+k_{2}k_{3}\left(k_{1}m_{31}^{-1}-k_{2}\right)z_{2}-k_{3}k_{2}k_{1}m_{31}^{-1}k_{1}z_{1}\right)+z_{6}^{T}\left(g_{2}+b_{2}u+z_{5}-k_{4}k_{4}z_{5}\right), \end{array}$$

where $Z={\left[z_{1},z_{2},z_{3},z_{4},z_{5},z_{6}\right]}^{T},K=diag\left[m_{31}^{-1}k_{1},k_{2}-k_{1}m_{31}^{-1},k_{3}-k_{2},-k_{3},k_{4},-k_{4}\right]$.

According to the controller (4), ${\dot{V}}_{1}$ can be rewritten as

$$\begin{array}{c} {\dot{V}}_{1}=-Z^{T}\hat{K}Z+z_{1}^{T}\left(m_{31}^{-1}-k_{1}m_{31}^{-1}k_{1}\right)z_{2}+z_{2}^{T}\left(I+k_{2}k_{1}m_{31}^{T}-k_{2}k_{2}\right)z_{3}-z_{1}^{T}k_{2}k_{1}m_{31}^{-1}k_{1}z_{3}, \end{array}$$

where $\hat{K}=diag\left[m_{31}^{-1}k_{1},k_{2}-k_{1}m_{31}^{-1},k_{3}-k_{2},\vartheta -k_{3},k_{4},\vartheta -k_{4}\right]$.

According to Young’s inequality, we can obtain

(9) $$\left\{\begin{array}{c} {z_{1}}^{T}\left(m_{31}^{-1}-k_{1}m_{31}^{-1}k_{1}\right)z_{2}\leq \frac{1}{4}z_{1}^{T}{\left(m_{31}^{-1}-k_{1}m_{31}^{-1}k_{1}\right)}^{T}\left(m_{31}^{-1}-k_{1}m_{31}^{-1}k_{1}\right)z_{1}+z_{2}^{T}z_{2}\\ z_{2}^{T}\left(I+k_{2}k_{1}m_{31}^{-1}-k_{2}k_{2}\right)z_{3}\leq \frac{1}{4}z_{2}^{T}z_{2}+z_{3}^{T}{\left(I+k_{2}k_{1}m_{31}^{-1}-k_{2}k_{2}\right)}^{T}\left(I+k_{2}k_{1}m_{31}^{-1}-k_{2}k_{2}\right)z_{3}\\ -z_{1}^{T}k_{2}k_{1}m_{31}^{-1}k_{1}z_{3}\leq \frac{1}{4}z_{1}^{T}{\left(k_{2}k_{1}m_{31}^{-1}k_{1}\right)}^{T}\left(k_{2}k_{1}m_{31}^{-1}k_{1}\right)z_{1}+z_{3}^{T}z_{3}. \end{array}\right.$$

We can obtain

$${\dot{V}}_{1}\leq -\sum_{j=1}^{6}z_{j}^{T}Q_{j}z_{j}.$$

${\dot{V}}_{1}$ can be expressed as

(10) $${\dot{V}}_{1}\leq -\varrho_{min}\sum_{j=1}^{6}z_{j}^{T}z_{j},$$

where $\varrho_{min}$ is the minimum eigenvalue of $Q_{j}$, j=1,2,⋯,6. When (5) is satisfied, $\varrho_{min}>0$.

According to (10), error $z_{j}$ is global asymptotic exponential stability, j=1,⋯,6, as is $e_{j}$. When $e_{3}=0,e_{6}=0$ are satisfied, $\xi_{3}=0,{\dot{\xi }}_{3}=0,{\dot{\xi }}_{2}=0$. $e_{1}\left(\xi_{1},{\dot{\xi }}_{2},\xi_{3},{\dot{\xi }}_{3}\right)=e_{1}\left(\xi_{1},0,0,0\right)=0$; we can obtain $\xi_{1}=\xi_{1d}$. According to the above analysis, the controller guarantees states converge asymptotically to the desired value. The proof is completed. □

### 3.2. Position Controller Based on Barrier-Lyapunov-like Method

When a quadrotor transports a payload, the swing of the payload not only severely weakens the stability of the quadrotor but also poses a risk of damage to the payload itself. Therefore, it is necessary to impose constraints on the swing angles of the payload. The swing angles satisfy α,β∈[−κ,+κ].

Considering the constraints of the swing angles of the payload, $e_{3}\left(\alpha ,\beta \right)$ satisfies $|e_{3}|\leq {\bar{f}}_{1}$, where ${\bar{f}}_{1}$ is the upper bound of $e_{3}$. Defining a variable $\sigma =e_{3}/{\bar{f}}_{1}$, we have σ∈(−1,1).

**Theorem** **2.** *Considering the QSLTS with underactuated state constraints, an anti-swing controller based on a barrier-Lyapunov-like method is designed. When the control parameters satisfy (12) and (13), the controller (11) guarantees the global asymptotic exponential stability of the position subsystem, while also satisfying state constraints:*

(11) $$\hat{u}=-{\left[\begin{array}{c} \frac{\partial f_{1}}{\partial q_{3}}b_{1}\\ b_{2} \end{array}\right]}^{-1}\left[\begin{array}{c} {\hat{u}}_{1}\\ {\hat{u}}_{2} \end{array}\right],$$

where ${\hat{u}}_{1}=\left(\frac{d}{dt}\frac{\partial f_{1}}{\partial q_{3}}\right)q_{4}+\frac{\partial f_{1}}{\partial q_{3}}g_{1}+\vartheta_{1}z_{4}+\left(\Gamma +k_{3}k_{2}-k_{3}k_{3}\right)z_{3}+k_{2}k_{3}\left(k_{1}m_{31}^{-1}-k_{2}\right)z_{2}-k_{3}k_{2}k_{1}m_{31}^{-1}k_{1}z_{1}+\frac{P\sigma z_{3}^{T}z_{3}}{{\bar{f}}_{1}\left(1-\sigma^{T}\sigma \right)},$ ${\hat{u}}_{2}=u_{2}$, $\Gamma =1+Plog(\frac{1}{1-\sigma^{T}\sigma })$.

We choose proper parameters, to satisfy

(12) $$\left\{\begin{array}{c} k_{i}>0,i=1,\cdots ,4\\ \vartheta_{1}>k_{3},\vartheta_{2}>k_{4}\\ eig({\overset{ˇ}{Q}}_{j})>0,j=1,\cdots ,6\\ eig(Q_{\sigma })\geq 0, \end{array}\right.$$

where

(13) $$\left\{\begin{array}{c} {\overset{ˇ}{Q}}_{1}=m_{31}^{-1}k_{1}-0.25K_{1}^{T}K_{1}-0.25K_{3}^{T}K_{3}\\ {\overset{ˇ}{Q}}_{2}=k_{2}-k_{1}m_{31}^{-1}-1.25\\ {\overset{ˇ}{Q}}_{3}=\Gamma \left(k_{3}-k_{2}\right)-K_{2}^{T}K_{2}-1-\frac{Pz_{2}^{T}k_{3}k_{2}z_{2}}{4{\bar{f}}_{1}\left(1-\sigma^{T}\sigma \right)}\\ {\overset{ˇ}{Q}}_{4}=\vartheta_{1}-k_{3}\\ {\overset{ˇ}{Q}}_{5}=k_{4}\\ {\overset{ˇ}{Q}}_{6}=\vartheta_{2}-k_{4}\\ Q_{\sigma }=Pk_{3}\frac{{\bar{f}}_{1}I-k_{2}}{\bar{f}\left(1-\sigma^{T}\sigma \right)}\\ K_{1}=m_{31}^{-1}-k_{1}m_{31}^{-1}k_{1}\\ K_{2}=I+\Gamma \left(k_{2}k_{1}m_{31}^{-1}-k_{2}k_{2}\right)\\ K_{3}=\Gamma k_{2}k_{1}m_{31}^{-1}k_{1}. \end{array}\right.$$

**Proof** **of** **Theorem** **2.** We choose the barrier-Lyapunov-like function

(14) $$V_{2}=V_{1}+\frac{P}{2}log\left(\frac{1}{1-\sigma^{T}\sigma }\right)z_{3}^{T}z_{3},$$

where *P* is a positive constant. Deriving both sides of (14), we obtain

(15) $$\begin{array}{c} {\dot{V}}_{2}=\sum_{i=1}^{6}z_{i}{\dot{z}}_{i}+\frac{z_{3}^{T}z_{3}\sigma^{T}}{{\bar{f}}_{1}\left(1-\sigma^{T}\sigma \right)}\dot{\sigma }+Plog\left(\frac{1}{1-\sigma^{T}\sigma }\right)z_{3}^{T}{\dot{z}}_{3}\\ =\sum_{i=1,j\neq 3}^{6}z_{i}{\dot{z}}_{i}+\Gamma z_{3}^{T}{\dot{z}}_{3}+\frac{Pz_{3}^{T}z_{3}\sigma^{T}\left(z_{4}-k_{3}\left(\bar{f}\sigma +k_{2}z_{2}\right)\right)}{{\bar{f}}_{1}\left(1-\sigma^{T}\sigma \right)}. \end{array}$$

In the light of the proof of Theorem 1, substituting (11) to (15), ${\dot{V}}_{2}$ can be rewritten as

(16) $$\begin{array}{c} {\dot{V}}_{2}=z_{1}^{T}m_{31}^{-1}\left(z_{2}-k_{1}z_{1}\right)+z_{2}^{T}\left(z_{3}-k_{2}z_{2}-{\dot{\lambda }}_{1}\right)+z_{3}^{T}\Gamma \left(z_{4}-k_{3}z_{3}-{\dot{\lambda }}_{2}\right)\\ +z_{4}^{T}\left({\dot{e}}_{4}-{\dot{\lambda }}_{3}\right)+z_{5}\left(z_{6}-k_{4}z_{5}\right)+z_{6}\left({\dot{e}}_{6}-{\dot{\lambda }}_{4}\right)+\frac{Pz_{3}^{T}z_{3}\sigma^{T}\left(z_{4}-k_{3}\left(\bar{f}\sigma +k_{2}z_{2}\right)\right)}{{\bar{f}}_{1}\left(1-\sigma^{T}\sigma \right)}\\ =-Z^{T}\bar{K}Z+z_{1}^{T}\left(m_{31}^{-1}-k_{1}m_{31}^{-1}k_{1}\right)z_{2}+z_{2}^{T}\left(I+\Gamma \left(k_{2}k_{1}m_{31}^{-1}-k_{2}k_{2}\right)\right)z_{3}-z_{1}^{T}\Gamma k_{2}k_{1}m_{31}^{-1}k_{1}z_{3}\\ -\frac{Pz_{3}^{T}z_{3}\sigma^{T}k_{3}\bar{f}\sigma }{{\bar{f}}_{1}\left(1-\sigma^{T}\sigma \right)}-\frac{Pz_{3}^{T}z_{3}\sigma^{T}k_{3}k_{2}z_{2}}{{\bar{f}}_{1}\left(1-\sigma^{T}\sigma \right)}, \end{array}$$

where $\bar{K}=diag\left[m_{31}^{-1}k_{1},k_{2}-k_{1}m_{31}^{-1},\Gamma k_{3}-k_{2},\vartheta -k_{3},k_{4},\vartheta -k_{4}\right]$.

As $\frac{Pz_{3}^{T}k_{3}k_{2}z_{3}}{{\bar{f}}_{1}\left(1-\sigma^{T}\sigma \right)}$ is positive, according to Young’s inequality, the following inequality is satisfied:

$$-\frac{Pz_{3}^{T}z_{3}\sigma^{T}k_{3}k_{2}z_{2}}{{\bar{f}}_{1}\left(1-\sigma^{T}\sigma \right)}\leq \frac{Pz_{3}^{T}k_{3}k_{2}z_{3}\sigma^{T}\sigma }{{\bar{f}}_{1}\left(1-\sigma^{T}\sigma \right)}+\frac{Pz_{3}^{T}z_{3}z_{2}^{T}k_{3}k_{2}z_{2}}{4{\bar{f}}_{1}\left(1-\sigma^{T}\sigma \right)}.$$

${\dot{V}}_{2}$ can be rewritten as

(17) $$\begin{array}{c} {\dot{V}}_{2}\leq -Z^{T}\bar{Q}Z-\frac{Pz_{3}^{T}k_{3}z_{3}\sigma^{T}\sigma }{\left(1-\sigma^{T}\sigma \right)}+\frac{Pz_{3}^{T}k_{3}k_{2}z_{3}\sigma^{T}\sigma }{{\bar{f}}_{1}\left(1-\sigma^{T}\sigma \right)}+\frac{Pz_{3}^{T}z_{3}z_{2}^{T}k_{3}k_{2}z_{2}}{4{\bar{f}}_{1}\left(1-\sigma^{T}\sigma \right)}\\ \leq -Z^{T}\bar{Q}Z-\frac{\left(Pz_{3}^{T}k_{3}\left(\bar{f}I-k_{2}\right)z_{3}\right)\sigma^{T}\sigma }{{\bar{f}}_{1}\left(1-\sigma^{T}\sigma \right)}+\frac{\left(Pz_{2}^{T}k_{3}k_{2}z_{2}\right)z_{3}^{T}z_{3}}{4{\bar{f}}_{1}\left(1-\sigma^{T}\sigma \right)}\\ \leq -Z^{T}\overset{ˇ}{Q}Z-\frac{z_{3}^{T}Q_{\sigma }z_{3}\sigma^{T}\sigma }{{\bar{f}}_{1}\left(1-\sigma^{T}\sigma \right)}, \end{array}$$

where $\overset{ˇ}{Q}=diag\left(\left[{\overset{ˇ}{Q}}_{1},{\overset{ˇ}{Q}}_{2},{\overset{ˇ}{Q}}_{3},{\overset{ˇ}{Q}}_{4},{\overset{ˇ}{Q}}_{5},{\overset{ˇ}{Q}}_{6}\right]\right)$.

The following inequality holds: $log\left(1/\left(1-\sigma^{T}\sigma \right)\right)\leq \sigma^{T}\sigma /\left(1-\sigma^{T}\sigma \right)$ when |σ|<1 is satisfied. ${\dot{V}}_{2}$ can be expressed as

(18) $${\dot{V}}_{2}\leq -{\overset{ˇ}{\varrho }}_{min}\sum_{j=1}^{6}z_{j}^{T}z_{j}-\rho_{min}log\left(\frac{1}{1-\sigma^{T}\sigma }\right)z_{3}^{T}z_{3},$$

where ${\overset{ˇ}{\varrho }}_{min}$ and $\rho_{min}$ are the minimum eigenvalues of ${\overset{ˇ}{Q}}_{i}$ and $Q_{\sigma }$, respectively; i=1,2,⋯6. Choosing $\Upsilon =min\{2{\overset{ˇ}{\varrho }}_{min},2\rho_{min}/P\}$, we obtain

(19) $${\dot{V}}_{2}\leq -\Upsilon V_{2}.$$

According to (19), error $z_{j}$ is global asymptotic exponential stability, j=1,⋯,6. The payload’s swing angles are exponentially stable within the constraints, as is $e_{j}$. □

**Note 1** *P* is a scale of constraints. P=0 means that there are no swing angle constraints on the system. According to (14), the controller (11) also ensures the stability of the system when P=0. On the other hand, to ensure $Q_{3}>0$, *P* should be chosen as a small positive constant.
**Note 2** In order to simplify the selection of parameters satisfying (5) and (13), $m_{31}$ is regarded as a constant matrix because the swing angles are small.
**Note 3** $eig(Q_{\sigma })>0$ infers ${\bar{f}}_{1}I>k_{2}$. Thus, there is a minimum boundary of swing angles.

### 3.3. Attitude Controller Design

In the inner–outer control structure, the outer controller ensures the stability of the position subsystem, while the inner one tracks the attitude angles extracted from the outer loop output, to guarantee the stability of the entire system.

The expected thrust force and attitude angles can be calculated:

(20) $$\begin{array}{c} F=\sqrt{U_{x}^{2}+U_{y}^{2}+{\left(U_{z}+\left(M_{q}+M_{l}\right)g\right)}^{2}}\\ \phi_{d}=asin\left(\left(U_{x}S_{\psi_{d}}-U_{y}C_{\psi_{d}}\right)/F\right)\\ \theta_{d}=asin\left(\left(U_{x}C_{\psi_{d}}+U_{y}S_{\psi_{d}}\right)/\left(FC_{\phi_{d}}\right)\right). \end{array}$$

Based on the attitude dynamics (2), we use a PD control approach. The attitude controller is designed as

(21) $$\tau =-K_{p}e_{\Phi }-K_{v}e_{\Omega }+W\left(\dot{\theta },\dot{\phi },\dot{\psi }\right),$$

where $W={\left[\left(I_{y}-I_{z}\right)\dot{\theta }\dot{\psi },\left(I_{z}-I_{x}\right)\dot{\phi }\dot{\psi },\left(I_{x}-I_{y}\right)\dot{\phi }\dot{\theta }\right]}^{T},\tau =\left[\tau_{x},\tau_{y},\tau_{z}\right]$, $e_{\Phi },e_{\Omega }$ are the angular error and angular velocity error, respectively; $K_{p},K_{v}\in R^{3\times 3}$ are positive diagonal constant matrices.

The controller (21) guarantees the attitude angles approach to the desired value exponentially.

## 4. Simulation and Results

In this section, several simulation results are provided, to illustrate the effectiveness of the proposed strategy. The parameters of the QSLTS were chosen as $M_{q}=1$ kg, $M_{l}=0.2$ kg, l=1 m, g=9.8 m/s${}^{2}$, $I_{x}=I_{y}=0.009$ kg/s${}^{2}$, $I_{z}=0.015$ kg/s${}^{2}$. The expected location was x=20 m, y=20 m, z=20 m. The constraints of the payload’s angles were $\left|\alpha \right|\leq 15^{\circ },\left|\beta \right|\leq 15^{\circ }$. The controller parameters were $k_{1}=diag\left(\left[0.5,0.5\right]\right),k_{2}=diag\left(\left[2.5,2.5\right]\right),k_{3}=diag\left(\left[20,20\right]\right),k_{4}=0.25,\vartheta_{1}=diag\left(\left[20.25,20.25\right]\right),\vartheta_{2}=2$, *P* = 0.05, satisfying (5) and (12). Results controlled by LQR and the Lyapunov method without constraints are provided as comparison.

**Figure 3:**
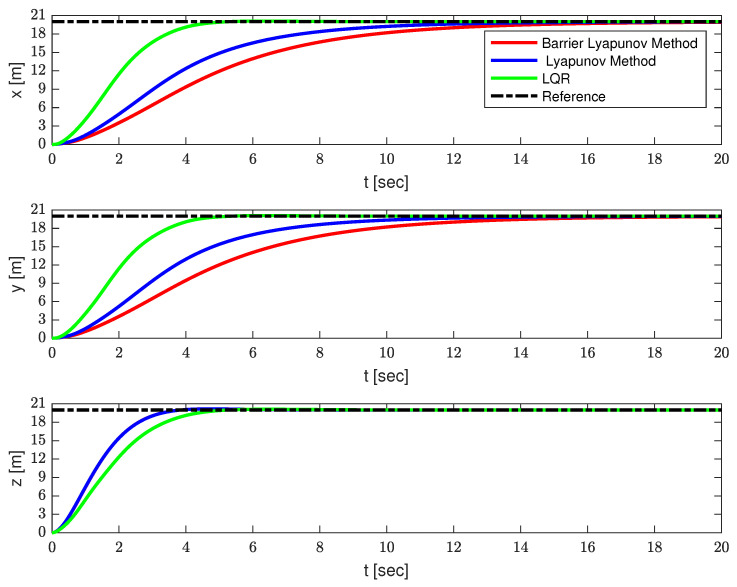
The position of the quadrotor.

**Figure 4:**
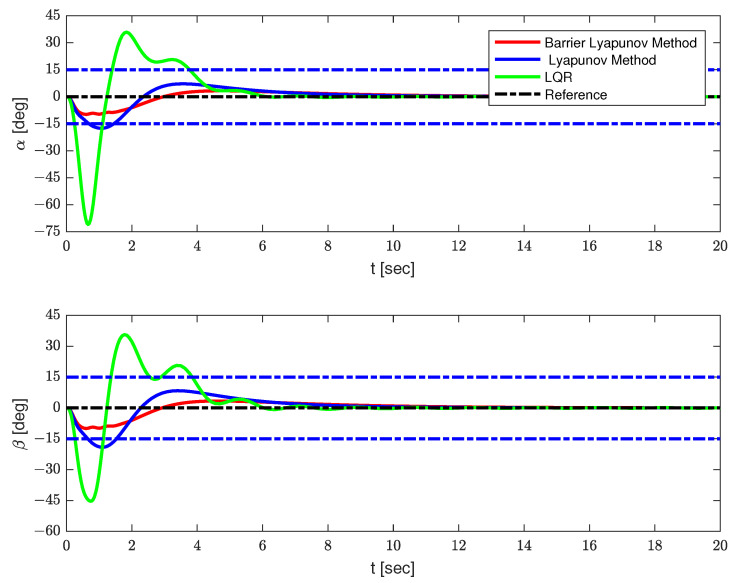
The position of the payload.

**Figure 5:**
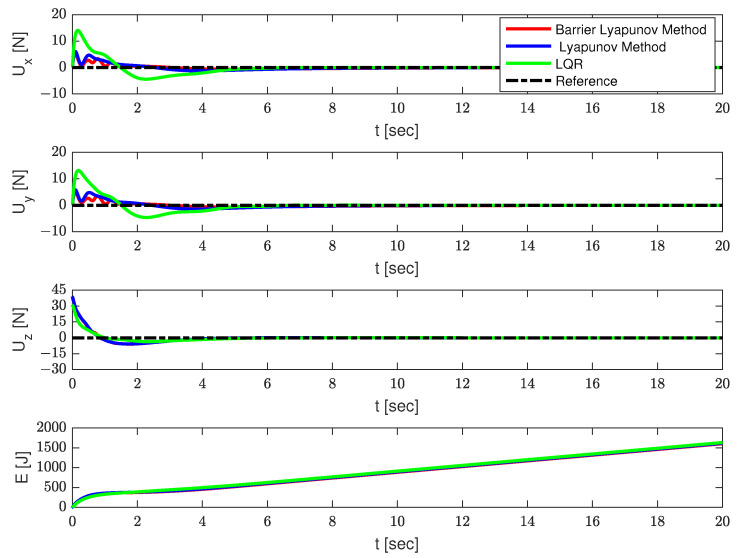
The force and energy consumption on the quadrotor.

The simulation results are shown in Figure 3, Figure 4 and Figure 5 and Table 2. The positions of the quadrotor are shown in Figure 3, the swing angles of the payload are presented in Figure 4, and Figure 5 illustrates the force acting on the drone and energy consumption during flying. The solid red lines represent the trajectories of the QSLTS, with the payload’s swing angle constraints controlled by the barrier Lyapunov method. The solid blue lines represent the states of the QSLTS without state constraints controlled by the Lyapunov method. The solid green lines represent the states of the QSLTS controlled by LQR. The dashed black lines are the desired values, and the dashed blue lines are the boundaries.

From Figure 3, Figure 4 and Figure 5, it can be observed that all the methods can drive the states to the desired position, while the LQR method exhibits the fastest response. However, it is important to note that the LQR controller failed to suppress the swing angles of the payload, with the maximum swing angle exceeding $70^{\circ }$. One of the control targets was to suppress the load’s swing, but LQR failed to constrain states efficiently, even changing parameters Q and/or R. Even after the quadrotor reached the desired position, the payload continued to swing. On the other hand, the control method based on the barrier Lyapunov function, as shown in Figure 4, successfully limited the swing angle of the payload within boundaries, at the expense of losing some speed. With no constraints limit, the swing angle of the payload far exceeded $15^{\circ }$. We also provide the control effort $E_{u}=\int_{0}^{20}\left(U_{x}^{2}+U_{y}^{2}+{\left(U_{z}+\left(M_{q}+M_{l}\right)g\right)}^{2}\right)dt$ as a comparison. The quantified results are shown in Table 2, where the number in bold font shows the optimum in each column. The proposed barrier Lyapunov method achieved satisfactory swing suppression both in overshoot and anti-swing settling time. With a small overshoot, the swing almost vanished within 6s. The proposed control method not only guarantees constraints on the payload swing angles but also results in lower energy consumption to accomplish the same tracking task. This demonstrates the effectiveness of the proposed method.

## 5. Conclusions

To address the issue of the swing of the payload in the QSLTS, an anti-swing control method for the QSLTS, based on the barrier Lyapunov function, was proposed in this paper. By considering the quadrotor and the payload as a whole, a dynamic model of the system was constructed, and an inner–outer loop control structure was explored, to ensure the stable control of the system. Based on the global coordinate transformation, the dynamics of the position subsystem were converted into a cascade form. Then, a Lyapunov function based on payload state constraints was designed, and a control law that ensures the global asymptotic stability of the position subsystem was constructed. Finally, an attitude controller was designed, to track the virtual outputs of the position controller. The simulation results demonstrated that the proposed controller not only ensures the quadrotor flying to the desired position but also limits the swing angle of the payload within boundaries. A comparison to the LQR control method and the unconstrained swing method was provided, to prove the effectiveness of the proposed approach.

In the future, the uncertainties, including the model uncertainties and external disturbance, will be taken into account. We plan to utilize a radial basis function neural network, to estimate them online, and to explore a Lyapunov-based controller, to ensure the stability of the whole system. These efforts have the potential to produce excellent results.

## Figures and Tables

**Table 1:** Parameters of the quadrotor-slung load transportation system.

Symbol	Description
O−XYZ	the global coordinate of the QSLTS
$O_{b}-X_{b}Y_{b}Z_{b}$	the local coordinate of the quadrotor
$I_{x},I_{y},I_{z}$	the moment of inertia along axes X, Y, and Z, respectively
$\tau_{z},\tau_{y},\tau_{z}$	roll, pitch, and yaw torques, respectively, acting on the quadrotor
α	the payload’s angle, with respect to its projection on the XOZ
β	the payload’s angle, with respect to its projection on the YOZ
$M_{q}$	the mass of the quadrotor
$M_{l}$	the mass of the payload
*l*	the length of the cable
$F_{i}$	the force generated by the *i*th rotor
*g*	the acceleration of gravity

**Table 2:** The quantified results of the QSLTS.

Methods	$t_{\mathrm{sx}}$[s]	$t_{\mathrm{sy}}$[s]	$t_{\mathrm{sz}}$[s]	$t_{s\alpha }$[s]	$t_{s\beta }$[s]	$|\alpha_{\max}|{[}^{\circ }]$	$|\beta_{\max}|{[}^{\circ }]$	$E_{u}^{*}\left[J\right]$
Barrier Lyapunov Method	15.01	14.96	**3.37**	5.66	5.69	**9.57**	**9.76**	**3203**
Lyapunov Method	13.96	13.69	**3.37**	5.95	5.96	17.52	19.11	3215
LQR	**4.44**	**4.44**	4.42	**5.52**	**5.67**	70.65	45.67	3266

* Total energy consumption during flying. The number in bold font is the optimum in each column.

## Data Availability

The data presented in this study are available on request from the corresponding author.

## References

[1] Ghadiri H., Emami M., Khodadadi H. (2021). Adaptive super-twisting non-singular terminal sliding mode control for tracking of quadrotor with bounded disturbances. Aerosp. Sci. Technol..

[2] Li C., Wang Y., Yang X. (2022). Adaptive fuzzy control of a quadrotor using disturbance observer. Aerosp. Sci. Technol..

[3] Perozzi G., Efimov D., Biannic J.M., Planckaert L. (2022). Using a quadrotor as wind sensor: Time-varying parameter estimation algorithms. Int. J. Control.

[4] Heidari H., Saska M. (2021). Trajectory planning of quadrotor systems for various objective functions. Robotica.

[5] Liu H., Tu H., Huang S., Zheng X. (2023). Adaptive predefined-time sliding mode control for QUADROTOR formation with obstacle and inter-quadrotor avoidance. Sensors.

[6] Zhou L., Xu S., Jin H., Jian H. (2021). A hybrid robust adaptive control for a quadrotor UAV via mass observer and robust controller. Adv. Mech. Eng..

[7] Um Y.C., Choi H.L. (2022). Integral gamma-sliding mode control for a quadrotor with uncertain time-varying mass and external disturbance. J. Electr. Eng. Technol..

[8] Liang X., Fang Y., Sun N., Lin H., Zhao X. (2021). Adaptive nonlinear hierarchical control for a rotorcraft transporting a cable-suspended payload. IEEE Trans. Syst. Man Cybern. A.

[9] Ameya R.G., Kamesh S. (2019). Nonlinear control of unmanned aerial vehicles with cable suspended payloads. Aerosp. Sci. Technol..

[10] Sreenath K., Lee T., Kumar V. Geometric control and differential flatness of a quadrotor UAV with a cable-suspended load. Proceedings of the IEEE Conference on Decision and Control.

[11] Shi D., Wu Z., Chou W. (2018). Harmonic extended state observer based anti-swing attitude control for quadrotor with slung load. Electronics.

[12] Santos M.A., Rego B.S., Raffo G.V., Ferramosca A. (2017). Suspended load path tracking control strategy using a tilt-rotor UAV. J. Adv. Transp..

[13] Xian B., Yang S. (2021). Robust tracking control of a quadrotor unmanned aerial vehicle-suspended payload system. IEEE Trans. Mechatron..

[14] Lv Z.Y., Li S., Wu Y., Wang Q.G. (2021). Adaptive control for a quadrotor transporting a cable-suspended payload with unknown mass in the presence of rotor downwash. IEEE Trans. Veh. Technol..

[15] Yang S., Xian B. (2020). Energy-based nonlinear adaptive control design for the quadrotor UAV system with a suspended payload. IEEE Trans. Ind. Electron..

[16] Yuan M., Chen Z., Yao B., Liu X. (2021). Fast and accurate motion tracking of a linear motor system under kinematic and dynamic constraints: An integrated planning and control approach. IEEE Trans. Control Syst. Technol..

[17] Wang X., Guo J., Tang S., Qi S., Wang Z. (2019). Entry trajectory planning with terminal full states constraints and multiple geographic constraints. Aerosp. Sci. Technol..

[18] He S., Hu C., Zhu Y., Tomizuka M. (2020). Time optimal control of triple integrator with input saturation and full state constraints. Automatica.

[19] Alkomy H., Shan J. (2021). Vibration reduction of a quadrotor with a cable-suspended payload using polynomial trajectories. Nonlinear Dyn..

[20] Liang X., Fang Y., Sun N., Lin H. (2018). Dynamics analysis and time-optimal motion planning for unmanned quadrotor transportation systems. Mechatronics.

[21] Yang T., Sun N., Fang Y. (2023). Neuroadaptive control for complicated underactuated systems with simultaneous output and velocity constraints exerted on both actuated and unactuated states. IEEE Trans. Neural Netw. Learn..

[22] Sun W., Su S.F., Wu Y., Xia J., Nguyen V.T. (2020). Adaptive fuzzy control with high-order barrier Lyapunov functions for high-order uncertain nonlinear systems with full-state constraints. IEEE Trans. Cybern..

[23] Laghrouche S., Harmouche M., Chitour Y., Obeid H., Fridman L.M. (2021). Barrier function-based adaptive higher-order sliding mode controllers. Automatica.

[24] Khadhraoui A., Zouaoui A., Saad M. (2023). Barrier Lyapunov function and adaptive backstepping-based control of a quadrotor UAV. Robotica.

[25] Hamed H., Ali S., Holger V., Mohamed D., Jose L.S. (2023). Safe navigation of a quadrotor UAV with uncertain dynamics and guaranteed collision avoidance using barrier Lyapunov function. Aerosp. Sci. Technol..

[26] Zhang H., Liu Y., Wang Y. (2021). Observer-based finite-time adaptive fuzzy control for nontriangular nonlinear systems with full-state constraints. IEEE Trans. Cybern..

[27] Ma H., Li H., Liang H., Dong G. (2019). Adaptive fuzzy event-triggered control for stochastic nonlinear systems with full state constraints and actuator faults. IEEE Trans. Fuzzy Syst..

[28] Hu W., Zhou Y., Zhang Z., Fujita H. (2021). Model predictive control for hybrid levitation systems of maglev trains with state constraints. IEEE Trans. Veh. Technol..

[29] Dong L., Yan J., Yuan X., He H., Sun C. (2019). Functional nonlinear model predictive control based on adaptive dynamic programming. IEEE Trans. Cybern..

[30] Yang G., Yao J., Dong Z. (2022). Neuroadaptive learning algorithm for constrained nonlinear systems with disturbance rejection. Int. J. Robust Nonlinear Control.

[31] Wu L.B., Park J.H., Xie X.P., Liu Y.J. (2021). Neural network adaptive tracking control of uncertain MIMO nonlinear systems with output constraints and event-triggered inputs. IEEE Trans. Neural Netw. Learn. Syst..

[32] Feng C., Wang Q., Liu C., Hu C., Liang X. (2020). Variable-structure near-space vehicles with time-varying state constraints attitude control based on switched nonlinear system. Sensors.

[33] Yu J., Dong X., Li Q., Lü J., Ren Z. (2022). Adaptive practical pptimal time-varying formation tracking control for disturbed high-order multi-agent Systems. IEEE Trans. Circuits Syst. I.

[34] Yang G. (2023). Asymptotic tracking with novel integral robust schemes for mismatched uncertain nonlinear systems. Int. J. Robust Nonlinear Control.

[35] Ding F., Huang J., Sun C., Ai Y., Yang C. (2023). Adaptive dynamic surface control for quadrotor-slung load transportation system with uncertainties. Sci. China Technol. Sci..

[36] Li X., Zhang H., Fan W., Wang C., Ma P. (2021). Finite-time control for quadrotor based on composite barrier Lyapunov function with system state constraints and actuator faults. Aerosp. Sci. Technol..

